# Low vaccination coverage for human papillomavirus disease among young men who have sex with men, France, 2019

**DOI:** 10.2807/1560-7917.ES.2021.26.50.2001965

**Published:** 2021-12-16

**Authors:** Giuseppina Ortu, Anne-Sophie Barret, Kostas Danis, Lucie Duchesne, Daniel Levy-Bruhl, Annie Velter

**Affiliations:** 1 Santé Publique France, Saint Maurice, France; 2 European Programme for Intervention Epidemiology Training (EPIET), European Centre for Disease Prevention and Control (ECDC), Stockholm, Sweden

**Keywords:** MSM, HPV, vaccination coverage

## Abstract

**Background:**

In France, human papillomavirus (HPV) vaccination has been recommended in 2016 for men who have sex with men (MSM) up to age 26 years.

**Aim:**

We aimed to estimate HPV vaccine coverage in 18–28 year-old MSM and identify uptake determinants.

**Methods:**

We collected data on socio-demographic characteristics, sexual behaviour, sexually transmitted diseases (STI) screening and vaccination uptake using a voluntary cross-sectional online survey conducted in 2019 targeting MSM. We calculated coverage of at least one dose of HPV vaccine and prevalence ratios (PR) of determinants with 95% confidence intervals (CI) using Poisson regression.

**Results:**

Of 9,469 respondents (age range: 18–28 years), 15% (95% CI: 14–16) reported being vaccinated for HPV. Coverage was significantly higher among MSM < 24 years (PR: 1.25; 95% CI: 1.13–1.39), with education level below university degree (PR: 1.12; 95% CI: 1.08–1.32), living in rural areas (PR: 1.21; 95% CI: 1.08–1.36), attending sex parties (PR: 1.12; 95% CI: 1.03–1.33), using HIV-related biomedical prevention methods (PR: 1.31; 95% CI: 1.12–1.54), with STI diagnosis (PR: 1.22; 95% CI: 1.08–1.38) and with hepatitis A or B vaccination (PR: 4.56; 95% CI: 3.63–5.81 vs PR: 3.35; 95% CI: 2.53–4.44).

**Conclusions:**

The HPV vaccination uptake among MSM in France was not satisfactory. It was higher among MSM benefitting from other vaccinations and biomedical preventive methods against HIV, suggesting a synergistic effect of the national preventive sexual health recommendations for MSM. Further efforts to improve HPV vaccination coverage targeting MSM are warranted.

## Introduction

Human papilloma virus (HPV) is a sexually transmitted infection that can lead to anal, genital and oral cancers. Approximately 4.5% of all new cancers cases per year worldwide (roughly 630,000) are attributable to HPV, with an estimated 8.6% in women and 0.8% in men [1]. Biological risk factors such as immunosuppression (e.g. in HIV patients [2]) and high-risk sexual behaviour [3,4] increase the risk of HPV infection and development of premalignant lesions and cancers. In men who have sex with men (MSM), genital and anal warts are common and incidence of HPV–related anal cancers is high in this population compared with heterosexual individuals [3,5]. Moreover, the higher incidence of HIV in MSM compared with general population and the increased risk of HPV in HIV-positive individuals, makes the MSM population even more at risk of HPV-related diseases.

There are 13 types of HPV considered at high risk of causing genital and anal premalignant lesions and cancers. Most frequently responsible for cancers are HPV 16/18 (87% of all anal cancers) [1]. Several vaccines have been developed to protect against HPV, among them the 4-valent recombinant vaccine and the most recent 9-valent vaccine [6]. It is estimated that the nine HPV types targeted by the 9-valent vaccines contribute to 95% of anal cancers [1]. In the last decade, most European countries have introduced HPV vaccination, generally via school based programmes or primary healthcare services [7], targeting pre-adolescent girls; other European countries have started vaccination in boys and girls, aiming at an universal coverage [6]. In some countries where vaccination was initiated only in girls, the vaccination programme was subsequently extended to groups at high risk of cancers related to the HPV types targeted by this vaccine, e.g. MSM.

Among a total of 352,000 estimated new cancer cases reported in France in 2015, an estimated 6,333 were attributable to HPV, of which 28% affected men [8]. France started the HPV immunisation programme in 2007 with the vaccination of girls at age 14 years and catch-up vaccination for girls and young women aged 15–23 years [9]. Later on, it was amended to include immunocompromised or asplenic individuals of both sexes and in 2016, extended to MSM up to the age of 26 years [10–14]. In 2017, a national strategy on sexual health (*Strategy Nationale de Sante Sexuelle 2017–2030* [15]) was launched, aiming to achieve 60% HPV vaccine coverage by 2023 and 80% HPV vaccine coverage by 2030 among adolescent girls. At that time, vaccination of boys was not considered cost-effective in the French context [13]; however, in 2019, the Collège de la Haute Autorité de santé, recommended extending HPV vaccination to this group, a recommendation that took effect in January 2021 [16].

Since 2016, MSM and other targeted groups can access vaccination via family physicians, sexual health practitioners and primary healthcare facilities; however, the 2016 High Council for Public Health (Haute Autorité de Santé) recommendation about HPV vaccination for men emphasised the need to offer the HPV vaccine free of charge to MSM also in sexual health centres (Centre Gratuit d’Information, de Dépistage et de Diagnostics, CeGIDD) [17,18], a recommendation that was put into practice in the same year [19].

To date, information on HPV vaccination uptake in MSM population in France is limited. The CeGIDD reported the number of HPV vaccination doses administered between 2016 and 2018 [19]; however, absence of data on the actual population of MSM accessing these centres made it impossible to estimate vaccination coverage in these facilities.

In 2019, a cross-sectional study targeting MSM, called the '*Enquête rapport au sexe*' (ERAS), was performed in France to describe the trends of the adoption of different methods of HIV prevention [20]. In this survey, questions related to the vaccination status and the uptake of screening for sexually transmitted infections were introduced. We aimed to estimate the HPV vaccination uptake among MSM aged between 18 and 28 years in France and to identify the socio-demographic factors and health determinants associated with vaccination in MSM since the recommendations were introduced.

## Methods

### Study overview and data collection

We used data from the ERAS cross-sectional study performed in France from 16 February to 31 March 2019. Details of the methodology used for this survey have been already published elsewhere [20].

In brief, a questionnaire was posted online on different networks such as gay dating (e.g. Grindr, Scruff, Hornet) or information websites (Têtu, Gayvox.fr, AgendaQ), generally targeting gay communities. In addition, the survey was distributed via other platforms such as Facebook and was available via links in websites accessed when using keywords related to homosexuality and encounters between men. Participation criteria were men 18 years and older.

### Inclusion criteria

We included in our analysis all participants that declared to be 'homosexual' or 'bisexual' or reported having at least one male sexual intercourse during their life. We selected for our analysis all MSM resident in metropolitan France and overseas French departments and all those up to the age of 28 years. This age was chosen to allow all MSM aged 26 years (maximum age for HPV vaccination eligibility) in 2016, who answered the questionnaire in 2019, to be included in the analysis.

The questionnaire gathered information on self-reported vaccination status for HPV, hepatitis A and hepatitis B ('yes' and among those answering 'yes': 'one dose', 'two or more doses' or 'I don’t know' options) and on the screening uptake for sexually transmitted infections. Information gathered on potential determinants of vaccination included: socio-demographic characteristics (origin, residence, financial status and education level); lifestyle such as circle of friends, number of sexual partners, attendance to saunas, bars, backrooms, outdoor meeting places or sex parties, use of web applications to date MSM; use of HIV prevention methods, namely condoms or biomedical HIV-related preventive measures (e.g. HIV pre-exposure prophylaxis (PrEP), treatment as prevention (TasP), treatment post-exposure (TPE)); and health determinants including perception of risk of being infected with an STI; uptake of screening for STI (syphilis, chlamydia, gonorrhoea, condyloma, hepatitis B and hepatitis C), and STI diagnoses.

### Statistical analysis

The questionnaire did not have the option to skip any of the questions; therefore, we did not have missing data. For numeric variables, we calculated means (and standard deviations) or medians (and ranges). We calculated the HPV vaccination coverage for at least one dose or for at least two doses, using as denominator those who answered 'yes', 'no' or 'unknown' to the relevant question. In this paper, we report the results from this analysis. The results of the sensitivity analysis where we excluded the unknown vaccination status from the denominator are in the Supplement.

To identify factors associated with HPV vaccination (at least one dose), we calculated prevalence ratios (PR) and 95% confidence intervals (95% CI). To calculate adjusted PR, we used Poisson regression. We included in the initial regression models all factors with a p value < 0.2 in the univariable analysis and those that could have been relevant for vaccination coverage based on the literature. We removed variables one at a time using the Wald test or the Akaike information criterium.

### Ethical statement

The study follows the ethical guidelines of the 1975 Helsinki Declaration. By clicking on a link or banner, the participant was directed to the survey site, which contained information about the aims and contents of the survey, terms of participation and data privacy. By clicking on a button containing the text “I have read and understood the information above”, the participant provided informed consent and was directed to the online questionnaire. The questionnaire was anonymous and self-administered and no IP addresses were collected. Santé Publique France review board approved the consent procedures on 20 March 2017 (DDPS-09).

## Results

Of the 24,308 respondents, 10,553 were between 18 and 28 years-old and within this population, 9,469 (median age: 23 years) met the inclusion criteria and were included in the analysis. Participants were from various regions and 22% lived in Île-de-France (Table 1). Among all participants, 73% (n = 6,883) defined themselves as homosexual and the remaining as bisexual, heterosexual or refused to answer; 70% (n = 6,598) had high education (Table 1).

**Table 1:** Description of the total MSM population, MSM vaccinated for HPV (any dose) and MSM not vaccinated (or with unknown vaccination status), aged 18–28 years, France, 2019 (n = 9,469)

Characteristics	All MSM (n = 9,469)		HPV-vaccinated MSM (n = 1,420)		MSM not vaccinated (or status unknown) (n = 8,049)		p value
	n	%	n	%	n	%	
**Age group (years)**							
18–19	1,686	17.8	276	19.4	1,410	17.5	0.040
20–24	4,974	52.5	759	53.5	4,215	52.4	
25–28	2,809	29.7	385	27.1	2,424	30.1	
**Residence area (region)**							
Île-de-France	2,116	22.4	315	22.2	1,801	22.4	NS
Overseas French departments	277	2.90	39	2.75	238	2.96	
Other regions	7,076	74.7	1,066	75.1	6,010	74.7	
**Commune of residence**							
Rural (< 2,000 inhabitants)	1,368	14.5	231	16.3	1,137	14.1	0.034
Urban	8,101	85.6	1,189	83.7	6,912	85.9	
**Origin (region)**							
Mainland France	8,606	90.9	1,277	89.9	7,329	91.1	NS
Overseas French departments	366	3.90	53	3.73	313	3.89	
Other countries	497	5.30	90	6.34	407	5.06	
**Financial status**							
Comfortable, getting by	4,859	51.3	746	52.5	4,113	51.1	NS
Struggling, soon to be in debt	4,610	48.7	674	47.5	3,936	48.9	
**Education level**							
College, baccalaureate	2,871	30.3	476	33.5	2,395	29.8	0.004
Higher education (university, Master, PhD)	6,598	69.7	944	66.5	5,654	70.2	
**Self-reported sexual orientation**							
Homosexual	6,883	72.7	1,040	73.2	5,843	72.6	NS
Bisexual or heterosexual or refused to answer	2,586	27.3	380	26.8	2,206	27.4	
**Attendance of saunas, bars, backrooms**							
No	5,140	54.3	747	52.6	4,393	54.6	NS
Yes	4,329	45.7	673	47.4	3,656	45.4	
**Attendance of outdoor meeting places (cruising areas)**							
No	7,868	83.1	1,143	80.5	6,725	83.6	0.005
Yes	1,601	16.9	277	19.5	1,324	16.5	
**Attendance of sex parties**							
No	8,667	91.5	1,245	87.7	7,422	92.2	< 10^−3^
Yes	802	8.47	175	12.3	627	7.79	
**Use of Internet sites or web applications for meeting MSM**							
No	2,365	25.0	345	24.3	2,020	25.1	NS
Yes	7,104	75.0	1,075	75.7	6,029	74.9	
**Circle of friends**							
Mainly homosexual	450	4.75	89	6.27	361	4.49	0.004
Homosexual and others (bisexual, heterosexual)	9,019	95.3	1,331	93.7	7,688	95.5	
**Vaccination uptake (self-declared)**							
Hepatitis A							
Not vaccinated	1,971	20.8	82	5.77	1,889	23.5	< 10^−3^
Vaccinated, any dose	4,050	42.8	1,274	89.7	2,776	34.5	
Unknown status	3,448	36.4	64	4.51	3,384	42.0	
Hepatitis B							
Not vaccinated	1,533	16.2	55	3.87	1,478	18.4	< 10^−3^
Vaccinated, any dose	5,102	53.9	1,328	93.5	3,774	46.9	
Unknown status	2,834	29.9	37	2.61	2,797	34.8	
**Screening uptake for any STI in the last 12 months (including HIV)**							
Not screened	3,973	42.0	473	33.3	3,500	43.5	< 10^−3^
Screened	5,496	58.0	947	66.7	4,549	56.5	
**Result of HPV or condyloma screening in the last 12 months**							
Not screened or negative	9,249	97.7	1,372	96.6	7,877	97.9	0.004
Positive	220	2.32	48	3.38	172	2.14	
**Result of any of the screenings performed**							
Not screened or negative	8,543	90.2	1,206	84.9	7,337	91.15	< 10^−3^
Positive	926	9.80	214	15.1	712	8.85	
**HIV status and use of PrEp**							
HIV-positive	81	0.90	23	1.62	58	0.72	0,004
HIV-negative and PrEP user	238	2.50	87	6.13	151	1.88	
HIV-negative and not PrEp user	6,258	66.1	940	66.2	5,318	66.1	
Unknown HIV status	2,892	30.5	370	26.1	2,522	31.3	
**Prevention methods used during the last sexual intercourse**							
No prevention methods	4,887	51.6	669	47.1	4,218	52.4	< 10^−3^
Condom use	3,227	34.1	494	34.8	2,733	34.0	
Biomedical prevention (TasP, PrEP, TPE)	404	4.27	121	8.52	283	3.52	
No sexual intercourse or practicing masturbation	951	10.0	136	9.58	815	10.1	
**Perception of risk of contracting STI other than HIV in the last 6 months**							
Low/moderate^a^	7,450	78.7	1,122	79.0	6,328	78.6	0.028
High^a^	562	5.90	102	7.18	460	5.71	
No sexual partner in the last 6 months	1,457	15.4	196	13.8	1,261	15.7	
**Number of occasional male sexual partners in the last 6 months**							
0–1	4,746	50.1	662	46.6	4,084	50.7	0.004
2 or more	4,723	49.9	758	53.4	3,965	49.3	

MSM: men who have sex with men; TasP: treatment as prevention; PrEP: pre-exposure prophylaxis; STI: sexually transmitted infection; TPE: treatment post-exposure. NS: not significant.

^a^ Scale: 0–7 = low/moderate; 8–10 = high.

Among the MSM, 15% (95% CI: 14–16; n = 1,420) reported being vaccinated against HPV, any dose, and 4.3% (95% CI: 3.9–4.7; n = 406) reported being vaccinated with two doses or more.

Of the MSM who reported being vaccinated with at least one HPV dose, 90% (n = 1,274) reported having been vaccinated for hepatitis A and 94% (n = 1,328) for hepatitis B; 67% (n = 947) reported having done at least one screening for STI including HIV; 3.4% (n = 48) reported a previous HPV infection or condyloma and 15% (n = 214) a positive diagnosis to any of the STI screenings performed in the last 12 months (other than HPV or condyloma, Tables 1 and 2). In terms of HIV, 1.6% (n = 23) reported to be HIV-positive, 66% (n = 940) were HIV-negative and did not use PrEP and 6.1% (n = 87) were HIV-negative and PrEP users; 47% (n = 669) of HPV-vaccinated MSM did not use any HIV prevention methods during their last anal sexual intercourse, 35% (n = 494) used condoms exclusively and 8.5% (n = 121) reported having used biomedical prevention methods (TasP, PrEP or TPE). The majority of the respondents also had a low or moderate perception of being at risk of contracting STI other than HIV (79%; n = 1,122), during the preceding 6 months and a majority (n = 1,224) had one or more sexual partners.

**Table 2:** Characteristics of MSM who declared being vaccinated for HPV (any dose) and associated factors age group 18–28 years, France, 2019 (n = 1,420)

Characteristics	HPV-vaccinated MSM			Univariable analysis			Multivariable analysis		
	n	%	95% CI	PR	95% CI	p	PR	95% CI	p
**Age group (years)**									
18–19	276	16.4	14.7–18.2	1.19	1.04–1.38	**0.014**	1.43	1.24–1.64	**< 0.001**
20–24	759	15.3	14.3–16.3	1.11	0.99–1.25	0.064	1.25	1.13–1.39	**< 0.001**
25–28	385	13.7	12.5–15.0	Ref			Ref		
**Residence area (region)**									
Île-de-France	315	14.9	13.4–16.5	Ref					
Overseas French departments	39	14.1	10.5–18.7	0.95	0.70–1.29	0.723			
Other regions	1,066	15.0	14.3–15.9	1.01	0.90–1.14	0.840			
**Commune of residence**									
Rural (< 2,000 inhabitants)	231	16.9	15.0–19.0	1.15	1.01–1.31	**0.033**	1.21	1.08–1.36	**0.001**
Urban	1,189	14.7	13.9–15.5	Ref			Ref		
**Origin (region)**									
France metropolitan	1,277	14.8	14.1–15.6	Ref					
Overseas French departments	53	14.5	11.2–18.5	0.98	0.76–1.26	0.851			
Other countries	90	18.1	15.0–21.7	1.22	1.01–1.48	**0.044**			
**Financial status**									
Comfortable, getting by	746	15.4	14.4–16.4	1.05	0.95–1.16	0.318			
Struggling, soon to be in debt	674	14.6	13.6–15.7	Ref					
**Education level**									
College, baccalaureate	476	16.6	15.3–18.0	1.16	1.05–1.28	**0.004**	1.12	1.08–1.32	**< 0.001**
Higher education (university, Master, PhD)	944	14.3	13.5–15.1	Ref			Ref		
**Self-reported sexual orientation**									
Homosexual	1,040	15.1	14.3–16.0	1.03	0.92–1.15	0.615			
Bisexual or heterosexual or refused to answer	380	14.7	13.4–16.1	Ref					
**Attendance of saunas, bars, backrooms**									
No	747	14.5	13.6–15.5	Ref					
Yes	673	15.6	14.5–16.7	1.07	0.97–1.18	0.169			
**Attendance of outdoor meeting places (cruising areas)**									
No	1,143	14.5	13.8–15.3	Ref					
Yes	277	17.3	15.5–19.2	1.19	1.06–1.34	**0.004**			
**Attendance of sex parties**									
No	1,245	14.4	13.6–15.1	Ref			Ref		
Yes	175	21.8	19.1–24.8	1.52	1.32–1.75	**< 0.001**	1.12	1.03–1.33	**0.019**
**Use of Internet sites or web applications for meeting MSM**									
No	345	14.6	13.2–16.1	Ref					
Yes	1,075	15.1	14.3–16.0	1.04	0.93–1.16	0.521			
**Circle of friends**									
Mainly homosexual	89	19.8	16.4–23.7	1.34	1.11–1.62	**0.003**			
Homosexual and others (bisexual, heterosexual)	1,331	14.8	14.0–15.5	Ref					
**Vaccination uptake (self-declared)**									
Hepatitis A									
Not vaccinated	82	4.2	3.4–5.1	Ref			Ref		
Vaccinated, any dose	1,274	31.5	30.0–32.9	7.56	6.09–9.39	**< 0.001**	4.56	3.63–5.81	**< 0.001**
Unknown status	64	1.9	1.5–2.4	0.45	0.32–0.62	**< 0.001**	0.71	0.49–1.03	0.069
Hepatitis B									
Not vaccinated	55	3.6	2.8–4.6	Ref			Ref		
Vaccinated, any dose	1,328	26.0	24.8–27.3	7.26	5.57–9.44	**< 0.001**	3.35	2.53–4.44	**< 0.001**
Unknown status	37	1.3	0.9–1.8	0.36	0.24–0.55	**< 0.001**	0.61	0.38–0.98	**0.041**
**Screening uptake for any STI in the last 12 months (including HIV)**									
Not screened	473	11.9	10.9–12.9	Ref					
Screened	947	17.2	16.3–18.3	1.45	1.31–1.60	**< 0.001**			
**Result of HPV or condyloma screening in the last 12 months**									
Not screened or negative	1,372	14.8	14.1–15.6	Ref					
Positive	48	21.8	16.9–27.8	1.47	1.14–1.90	**0.003**			
**Result of any of the screenings performed**									
Not screened or negative	1,206	14.1	13.4–14.9	Ref			Ref		
Positive	214	23.1	20.5–25.9	1.64	1.44–1.86	**< 0.001**	1.22	1.08–1.38	**0.002**
**HIV status and use of PrEp**									
HIV-positive	23	28.4	19.7–39.1	1.89	1.33–2.68	**< 0.001**			
HIV-negative and PrEP user	87	36.6	30.7–42.9	2.43	2.04–2.91	**< 0.001**			
HIV-negative and not PrEp user	940	15.0	14.2–15.9	Ref					
Unknown	370	12.8	11.6–14.1	0.85	0.76–0.95	**0.005**			
**Prevention methods used during the last sexual intercourse**									
No prevention methods	669	13.7	12.8–14.7	Ref			Ref		
Condom use	494	15.3	14.1–16.6	1.12	1.00–1.25	**0.041**	1.03	0.93–1.13	0.562
Biomedical prevention (TasP, PrEP, TPE)	121	30.0	25.7–34.6	2.19	1.86–2.58	**< 0.001**	1.31	1.12–1.54	**0.001**
No sexual intercourse or practicing masturbation	136	14.3	12.2–16.7	1.04	0.88–1.24	0.616	1.05	0.90–1.22	0.538
**Perception of risk of contracting STI other than HIV in the last 6 months**									
Low/moderate^a^	1,122	15.1	14.3–15.9	Ref					
High^a^	102	18.2	15.2–21.6	1.21	1.00–1.45	**0.046**			
No sexual partners in the last 6 months	196	13.5	11.8–15.3	0.89	0.78–1.03	0.116			
**Number of occasional male sexual partners in the last 6 months**									
0–1	662	14.0	13.0–15.0	Ref					
2 or more	758	16.1	15.0–17.1	1.15	1.05–1.27	**0.004**			

CI: confidence interval; MSM: men who have sex with men; TasP: treatment as prevention; PR: prevalence ratio; PrEP: pre-exposure prophylaxis; Ref: reference value; STI: sexually transmitted infection; TPE: treatment post-exposure.

^a^ Scale: 0–7 = low/moderate; 8–10 = high.

Prevalences are shown as row percentages (totals in Table 1). For the multivariable analysis, we show only prevalence ratios related to variables included in the multivariable model and remaining significant in the final accepted model. Significant values are shown in bold.

In the multivariable analysis, HPV vaccination coverage was significantly higher among MSM younger than 24 years (age 18–19 years: PR = 1.4; 95% CI: 1.2–1.4; and age 20–24 years: PR = 1.3; 95% CI: 1.1–1.4), among those with an education level below university degree (PR = 1.1; 95% CI: 1.1–1.3), those living in rural areas (PR = 1.2; 95% CI: 1.1–1.4) and those attending sex parties (PR = 1.1; 95% CI: 1.0–1.3) (Table 2). The MSM who had benefited from biomedical HIV prevention methods during the last anal intercourse (PR = 1.3; 95% CI: 1.1–1.5), those that declared having at least one STI diagnosis in the previous 12 months (PR = 1.2; 95% CI: 1.1–1.4) and those vaccinated for hepatitis A or B (PR = 4.6; 95% CI: 3.6–5.8 and PR = 3.4; 95% CI: 2.5–4.4, respectively) were also more likely to be vaccinated for HPV than others (Table 2). We did not observe collinearity or interaction between age and education or age and any other variable (data not shown).

We conducted the above analysis considering two doses of HPV vaccination. However, the number of observations was very small and not sufficiently powered for a meaningful analysis. 

Results from the sensitivity analysis (see Supplement), where we excluded the unknown vaccination status from the denominator, were consistent with the main results as presented above, namely associations between higher HPV vaccination and younger age, residence in rural area, lower education status and reporting other vaccinations such as hepatitis A and hepatitis B. Living in regions other than Île-de-France was positively associated with HPV vaccination, possibly reflecting residence in rural areas. The sensitivity analysis diverged from the presented analysis for other factors such as association with the use of biomedical HIV prevention methods and attendance at sex parties as these variables were significant in the univariable analysis, but did not remain significant in the multivariable analysis.

## Discussion

Our analysis indicated an HPV vaccination coverage (any dose) of 15% among all MSM aged 18–28 years, indicating an unsatisfactory level of vaccination in this high-risk group. A similar HPV coverage (any dose of 18% among MSM was reported in an online survey performed in 2018 in France [21]. HPV vaccination of girls has also been reported to be as low as 24% for two doses in France [22]. This level of immunity is not high enough to offer protection to boys, and MSM do not directly benefit from vaccination of girls through herd immunity. HPV vaccination for boys aged 11–14 years (with a catch-up vaccination up to age 19 years) will be implemented in France in 2021 [16]. However, even with universal vaccination, protection from HPV cancers of future MSM populations will take more than a decade to take effect, indicating that MSM remain highly vulnerable to HPV-related diseases. This low HPV coverage in MSM in France highlights the need to increase awareness of the impact of HPV infections specifically in MSM and the urgency to invest resources to improve vaccination uptake in this vulnerable population.

In this study, we also observed that only 4.3% among all respondents reported having received two doses or more of HPV vaccine. When given at young age, two doses of HPV vaccine are recommended; if vaccination is started at age 15 years or later, a three-dose regime should be followed to ensure optimal effectiveness [23]. Our results raise concerns about the impact of this vaccination in MSM in the long term, considering the very low level of uptake of the second dose. In another study among MSM in France, the vaccine uptake for three doses was estimated at 3.4%, which is consistent with our results [21]. Similar unsatisfactory HPV vaccination coverage for two doses has been reported in adolescent girls in France, with 24% coverage in 2018 [22]. Further studies are needed to explore access to subsequent doses of vaccination following the first one.

Information about HPV vaccination coverage in the MSM population in other countries is limited, either because vaccination targeting this group has only recently begun, e.g. in Canada [24] or Ireland [25], or because coverage has been calculated in specific MSM groups, for instance MSM accessing sexual health clinics for other medical issues, such as in the United Kingdom (UK) [26]. Some data are available from the United States (US), where HPV vaccination among MSM attending gay venues was reported at a level similar to our results (17%) [27].

Our findings indicate that higher HPV vaccination in MSM was associated with younger age. Association with younger age has already been documented in previous studies in the US [27–29]. However, we should take into consideration that convenience sampling methods such as ERAS are known to induce participation bias linked to age [30–32]. Participating in such surveys involves spending time at gay cruising or dating websites, and younger age groups, more sexually active than older groups, are likely to spend more time on such websites. Younger participants are possibly also the most self-assured about their own homosexuality, probably more informed about preventive health related to sexual practices and more keen to answer surveys on sexual health.

We saw that higher HPV vaccination in MSM was positively associated with lower education level, a result in contrast to what has been observed for instance in the US [33]. In France, the association between educational level and adhesion to vaccination varies according to studies and vaccines. In a 2016 study on vaccine perception in France [34], a greater proportion of vaccine positivity was reported among parents with a lower educational level. Another study on determinants of HPV vaccination reported a lower uptake among girls who had parents with higher educational level, but the opposite was observed among women aged 15–25 years [35]. In our analysis, it is unlikely that the apparent association between higher vaccination and lower education level was the confounding effect of age, as we adjusted for age.

Similarly to our results, higher vaccine uptake in MSM living in rural settings was also reported in a 2016 pilot study in the UK, but no explanations for this finding were provided [26]. Conversely, results in the US indicated higher vaccine uptake in urban settings [28,36]. We cannot explain our findings in France, especially considering that the CeGIDD are mostly located in hospitals in urban areas and these centres are where MSM receive medical support related to sexual health, including recommended vaccinations.

Diagnosis of any STI in the last 12 months and vaccinations for hepatitis A and B (recommended to MSM in France [37,38]) were positively associated with HPV vaccination. This association may reflect a contact with sexual healthcare personnel who recommend a package of interventions, including HPV vaccination, for MSM following disclosure of their homosexuality. This hypothesis is corroborated by a 2016 French study reporting that general practitioners advised for example vaccination against hepatitis A virus, to MSM who disclosed their sexual orientation [39]. These findings concur with the higher vaccination coverage in MSM using HIV-preventive biomedical interventions such as TasP, PrEP or TPE in our study. A positive association between HPV vaccination and implementation of medical preventive measures or provision of recommendations targeting MSM was also reported in Australia [40]. However, our data do not allow to conclude whether this association reflects a specific profile of certain MSM more prone to adopt various prevention measures regarding risks attached to their sexual practices, the linked offer of vaccination with other prevention measures by specialised heath structures offering care to MSM or a combination of both. Of note, in France, PrEP is provided free of charge and the first consultation before PrEP prescription implies a medical check-up that should include a recommendation on vaccinations (hepatitis A, hepatitis B and HPV) [41].

We also identified a positive association between attendance at sex parties and HPV vaccination, although in the sensitivity analysis, this association did not remain significant. Attendance at sex parties where group sex can occur has previously been associated with higher risk of STI and with use of PrEP in HIV-negative MSM [42]. MSM engaging in risky sexual behaviours may be more proactive in accessing sexual health clinics and adopting sexual health-related MSM-specific preventive measures, including HPV vaccination. On the other hand, men who have not yet declared their homosexuality or assumed their sexual orientation are potentially unaware of HPV vaccination, probably because they are not linked to the MSM community; however, the risk of being infected with HPV may increase once they have decided and accepted their sexual orientation.

Based on these results it would be important to identify ways to reach adolescent males after sexual debut and ensure that HPV prevention measures are known and adopted. It should be considered that while we cannot rule out any bias caused by the classification of men with unknown status as unvaccinated in the main analysis, these discrepancies between the main and the sensitivity analyses may be due to the high number of unknown answers and the consequent elimination of more than half of the available observations in the sensitivity analysis, which may have decreased the power and therefore the possibility to obtain significant associations, apart from the strongest.

The main strength of our study was that we used a large dataset with a diversified the MSM population in terms of socio-demographic characteristics.

Our study had some limitations. Firstly, men assertive about their homosexuality, more interested in sexual health prevention or affected by STI may have been more likely to complete the online questionnaire, suggesting that participants might not be representative of the whole MSM population and introducing a potential selection bias [32]. This bias could have led to an overestimated vaccination uptake compared with what we could have observed if even those not affected by STI or less interested had participated; therefore the actual vaccine coverage may be even lower than estimated in our data. However, the use of different types of platforms to advertise this survey may have helped recruit individuals with different background, interests, health status, lifestyle and geographical setting and may have limited this potential bias. Secondly, vaccination status and the number of vaccination doses were self-reported. Self-reported vaccination status may be influenced by recall bias, especially regarding the number of doses received, leading to a potential underestimation of the vaccine uptake for at least two doses.

## Conclusions

The low HPV vaccination coverage among MSM raises concerns about the possibility of decreasing the burden of HPV-related lesions and cancers in this population in France. Lower uptake was associated with older age, higher education level and living in urban areas. Higher vaccine uptake was observed among MSM benefitting from other vaccinations and biomedical preventive methods against HIV. It is essential to continue the vaccination programme targeting MSM and to invest further resources to improve awareness among MSM of the risk of HPV-related diseases and the importance of vaccination. In addition, it would be useful to establish a surveillance strategy to periodically estimate vaccination uptake in this population and to assess the impact of this vaccination approach by monitoring the prevalence of HPV infections in the MSM population. Improvement of HPV vaccination coverage in MSM will eventually contribute to the national HPV vaccination objectives.

## Supplementary Data

150000 Supplement
