# Supplementary material for: Low vaccination coverage for human papillomavirus disease among young men who have sex with men, France, 2019
Source: Euro Surveill. 2021 Dec 16;26(50):2001965. doi: 10.2807/1560-7917.ES.2021.26.50.2001965 (PMC8728497; doi:10.2807/1560-7917.ES.2021.26.50.2001965)
Supplement: Supplement [file 20-01965_ORTU_Supplement.pdf]

This supplementary material is hosted by *Eurosurveillance* as supporting information alongside the article "*Low vaccination coverage for human papillomavirus disease among men who have sex with men, France, 2019*" on behalf of the authors who remain responsible for the accuracy and appropriateness of the content. The same standards for ethics, copyright, attributions and permissions as for the article apply. Supplements are not edited by *Eurosurveillance* and the journal is not responsible for the maintenance of any links or email addresses provided therein.

Supplementary material

Characteristics of MSM declaring being vaccinated for HPV and associated factors. Unknown vaccination status has been excluded from the denominator in this analysis.

	N	%	95% CI	
**MSM HPV vaccinated**	**1420**	**32.4**	**31.0**	**33.8**
Non vaccinated	2966	66.2	66.2	69.0

	Univariable analysis				Multivariable analysis			
**Characteristics**	**PR**	**95% CI**		**P**	**PR**	**95% CI**		**P**
**Age group**								
18-19	1.72	1.52	1.95	0.000	1.44	1.30	1.60	**0.000**
20-24	1.32	1.19	1.47	0.000	1.28	1.17	1.40	**0.000**
25-28	ref				ref			
**Residence area (region)**								
Île-de-France	ref				ref			
Overseas French departments	1.29	0.99	1.68	0.057	1.10	0.90	1.35	0.417
Other regions	1.09	0.98	1.21	0.118	1.11	1.01	1.23	**0.024**
**Size of commune of residence (inhabitants)**								
Rural	1.30	1.17	1.45	0.000	1.14	1.05	1.25	**0.003**
Urban	ref				ref			
**Origin**								
France metropolitan	ref							
Overseas French departments	1.32	1.07	1.63	0.009				
Other countries	1.14	0.96	1.35	0.133				
**Financial status**								
Comfortable, getting by	0.97	0.89	1.05	0.428				
Struggling, soon to be in debt	ref							
**Education level**								
College, baccalaureate	1.42	1.31	1.55	0.000	ref			
Higher education (university, master, PhD)	ref				0.80	0.74	0.86	**0.000**
**Self-reporting sexual orientation**								
Homosexual	0.89	0.81	0.98	0.014				

Bisexual or heterosexual or refused to answer	ref							
**Attendance of saunas, bars, backrooms**								
No	ref							
Yes	0.92	0.84	1.00	0.047				
**Attendance of outdoor meeting places (cruising areas)**								
No	ref							
Yes	1.12	1.01	1.25	0.036				
**Attendance of sex parties**								
No	ref							
Yes	1.28	1.13	1.44	0.000				
**Use of internet sites or web applications for meeting MSM**								
No	ref				ref			
Yes	0.92	0.84	1.02	0.110	0.93	0.86	1.00	0.066
**Circle of friends**								
Mainly homosexual	1.07	0.90	1.27	0.475				
Homosexual and others (bisexuals, heterosexuals)	ref							
**Vaccination uptake**								
Hepatitis A								
Non vaccinated	ref				ref			
Vaccinated, any dose	11.32	9.14	14.03	0.000	6.60	5.22	8.34	**0.000**
Unknown status	2.91	2.13	3.96	0.000	2.34	1.69	3.26	**0.000**
Hepatitis B								
Non vaccinated	ref				ref			
Vaccinated, any dose	10.07	7.76	13.08	0.000	3.72	2.83	4.89	**0.000**
Unknown status	2.95	1.99	4.38	0.000	1.83	1.22	2.76	**0.003**
**HIV Status**								
HIV negative								
HIV positive	1.55	1.15	2.09	0.004				
Status unknown	1.17	1.06	1.29	0.002				
**Screening uptake for any of the STIs in the last 12 months (including HIV)**								
No screened	ref							
Screened	0.96	0.88	1.05	0.395				
**Positive to HPV or condyloma screening**								
Negative	ref				ref			
Positive	0.79	0.62	1.02	0.068	0.79	0.62	1.00	0.052
**Positive to any of the screenings performed**								
Negative	ref							
Positive	1.07	0.95	1.20	0.277				
**HIV status and use of PrEp users**								
HIV positive	1.60	1.19	2.16	0.002				

HIV negative and PrEP user	1.66	1.42	1.95	0.000	
HIV negative and non-PrEp user	ref				
Unknown	1.21	1.10	1.33	0.000	
**Prevention methods used during the last sexual intercourse**					
No prevention methods	ref				
Condoms use	1.05	0.96	1.16	0.301	
Biomedical prevention (TasP, PrEP, TPE)	1.48	1.28	1.71	0.000	
Not sexual intercourse or practicing masturbation	1.31	1.13	1.51	0.000	
**Perception of risk of contracting STIs other than HIV in the last 6 months**					
Low /Moderate	ref				
High	0.95	0.80	1.13	0.570	
No sexual partners in the last 6 months	1.13	1.00	1.28	0.048	
**Number of occasional male sexual partners in the last 6 months**					
0-1	ref				
2 or more	0.96	0.88	1.05	0.370	
